# Microcystin-LR does not induce alterations to transcriptomic or metabolomic profiles of a model heterotrophic bacterium

**DOI:** 10.1371/journal.pone.0189608

**Published:** 2017-12-14

**Authors:** Robbie M. Martin, Stephen P. Dearth, Gary R. LeCleir, Shawn R. Campagna, Elizabeth M. Fozo, Erik R. Zinser, Steven W. Wilhelm

**Affiliations:** 1 Department of Microbiology, University of Tennessee, Knoxville, Tennessee, United States of America; 2 Department of Chemistry, University of Tennessee, Knoxville, Tennessee, United States of America; CEA-Saclay, FRANCE

## Abstract

Microcystins are secondary metabolites produced by several freshwater, bloom-forming cyanobacterial species. Microcystin-producing cyanobacteria co-occur with a complex community of heterotrophic bacteria. Though conflicting, studies suggest that microcystins affect the physiology of heterotrophic bacteria by inducing oxidative stress and increasing cell envelope permeability. Based on these observations, we hypothesized that exposure to microcystin should induce differential expression in genes responding to oxidative and envelope stress and trigger shifts in metabolite pools. We tested this hypothesis by exposing *Escherichia coli* MG1655 to 1 and 10 mg/L microcystin-LR and monitored global changes to gene expression, cellular metabolite pools, and lipid composition using RNA-sequencing and UPLC-MS. Contrary to reported studies, we observed no evidence that microcystin-LR induced oxidative or cell envelope stress in *E*. *coli* under the tested conditions. Our results suggest a potential difference in mechanism by which microcystin-LR interacts with heterotrophic bacteria *vs*. cyanobacteria.

## Introduction

Microcystins are secondary metabolites produced by a number of freshwater, bloom-forming cyanobacteria that include species from the genera *Microcystis*, *Anabaena*, *Planktothrix*, *Oscillatoria*, and *Nostoc*. However, *Microcystis* spp. are typically the most common and widespread producers of greatest concern [1, 2]. The presence of microcystins during a harmful cyanobacterial bloom greatly increases the potential for ecologic harm, economic loss, and the threat to public health [3–5].

Microcystin is an enigmatic metabolite. It is a nitrogen- and energy-expensive molecule to produce [6], suggesting that it must provide some advantage to producers; however, many strains of the aforementioned genera are genetically incapable of making the toxin [7]. Blooms are often comprised toxic and non-toxic strains, with successional replacement of one type for the other occurring over the course of the bloom [8–10]. While a number of physiological and ecological functions of microcystin have been proposed, including allelopathy, cell signaling, cell-wide metabolism regulation, and protein stabilization during periods of oxidative stress [11–14], no intracellular function has been clearly demonstrated as of yet.

Microcystin is a cyclic peptide composed of seven amino acids. Over 200 congeners have been identified and they differ primarily by amino acids incorporated into the ring at positions 2 and 4 and by methylation of the ring at various positions [1, 15]. Microcystin-producing cyanobacteria co-occur with a complex and dynamic community of free-living and epibiotic heterotrophic bacteria [16–18]. An immediate ecological question then arises: how does microcystin affect the physiology of heterotrophic bacteria co-occurring with a bloom. A handful of studies have directly addressed this question using purified microcystins [19–25]. Results have been varied and sometimes conflicting. The earliest study reported that microcystin had no effect on unspecified Gram-negative and Gram-positive bacteria [19]. Lahti *et al*. [21] observed variable effects ranging from stimulatory to inhibitory when fractionated extracts containing microcystin-RR (MCRR; arginine incorporated into positions 2 and 4) were applied to bioluminescent bacteria and *Pseudomonas putida*. Two additional studies suggested microcystin had little effect on growth. In one, neither microcystin-LR (MCLR; leucine and arginine incorporated into positions 2 and 4, respectively) or MCRR at up to 8 mg/L produced growth inhibition in *Micrococcus luteus*, *Bacillus cereus*, *B*. *subtilis*, *Aeromonas hydrophila* or *Escherichia coli* [22], while in the other, no growth inhibition was observed in either *Bacillus* sp. or *P*. *aeruginosa* using an unspecified concentration of microcystin [23]. In contrast, Valdor and Aboal [24] found that *E*. *coli* growth was inhibited by MCLR concentrations of 5 mg/L and higher and by MCRR and microcystin-YR (MCYR; tyrosine and arginine incorporated into positions 2 and 4, respectively) at concentrations of 12.5 mg/L and higher. A more recent study showed species-dependent growth inhibition of isolates from lakes in Portugal using microcystin-LR, -RR, and -YR at concentrations as low as 1 μg/L [25].

A perceived limitation in the above studies is that growth was the only phenotype measured, providing little insight into possible modes of activity by microcystin. Two studies have investigated this question in greater detail. One reported that 2.5 mg/L of MCRR permeabilized the membrane of *E*. *coli* in a manner similar to 2.5 mg/L of polymyxin B nonapeptide and had a rapid and dramatic synergism with five different hydrophobic antibiotics: MCRR plus the antibiotic reduced the minimum inhibitory concentration of the antibiotic by ~30-60-fold relative to the same concentration of antibiotic alone [26]. In addition, MCRR caused release of periplasmic proteins by disrupting the outer membrane, while leaving the cytoplasmic membrane unaffected. In a separate study, Yang *et al*. [27] exposed *E*. *coli* to MCRR concentrations of 1, 5, 10, and 15 mg/L. Superoxide dismutase and catalase activities were higher, and growth rates lower, in a dose-dependent manner at concentrations ≥ 5 mg/L. Glutathione, reactive oxygen species (ROS), lipid peroxidation, and glutathione reductase activity increased in a dose-dependent manner at concentrations ≥ 10 mg/L.

While components of these reports are conflicting, they suggest that microcystin has potential to affect the physiology of heterotrophic bacteria in a congener- and species-dependent manner. Despite clear ecological implications, scant research has focused on this question, and none have employed methods that monitor cell-wide responses. The objective of this study was to evaluate the cellular response of a model heterotrophic bacterium exposed to ecologically relevant concentrations of microcystin. Towards this objective, *E*. *coli* MG1655 was exposed to 1 mg/L and 10 mg/L MCLR for one hour. During this time, we measured changes to gene expression, cellular metabolite pools, and lipid composition using RNA-seq and UPLC/MS. Our results suggest that even very high concentrations of MCLR have minimal impact on the physiology of *E*. *coli* and do not support the findings of several earlier studies.

## Material and methods

### Growth conditions and treatments

*E*. *coli* K12 strain MG1655 was grown at 37°C with shaking at 200 rpm in M9 minimal medium [28] modified with the addition of 4 g/L glucose and 1 mg/L thiamine HCl. Growth of cultures was determined by measuring optical density (OD) at 600 nm (Genysis 20, Thermo Electron Corp.). OD of all cultures was measured at experimental time points 0 and 60 min. Growth rate μ (hr^-1^) for each culture was calculated from these two OD readings. Time points 0 and 60 min corresponded to early and mid-exponential growth phase, respectively, as determined in preliminary experiments.

Microcystin-LR (Cayman Chemical Company, Ann Arbor, MI) dissolved in 100% ethanol was added to treatment cultures at concentrations of 1 mg/L and 10 mg/L. A solvent only treatment was added as a control. Final concentration of ethanol in all cultures was 1% (v/v). *E*. *coli* was exposed to MCLR for a period of 1 h. Samples for RNA-seq and metabolite and lipid composition analysis were taken at time 0 and every 15 min thereafter.

MCLR was used in this study because of its environmental relevance: it is typically the most common and abundant congener [1, 29]. Microcystin concentrations encountered during blooms are highly variable, but are typically less than 1 mg/L. However, concentrations as high as 25 mg/L have been reported in extremely dense blooms [1]. The concentrations used in our experiments were chosen to represent the high end of an environmentally relevant range (1 mg/L) as well as a high concentration (10 mg/L) that was similar to those used in other studies [22, 24, 27]. This allowed our results to be more directly comparable to those of previous works.

All experiments were performed in biologic triplicate. For RNA-sequencing and lipid samples, an overnight culture was started in M9 medium using a single colony picked from Lysogeny Broth (LB) agar plates. A 450-mL master culture in a 2-L flask was started by diluting the overnight culture 1:299 to allow for a period of rapid growth adequate to relieve the cells of the generalized stress response induced by their former overnight stationary phase status [30, 31]. To time the start of treatments with the early exponential growth phase, the master culture was grown to an OD_600_ ~ 0.30, then divided into three 132-mL treatment cultures grown in 500-mL flasks. At T_0_, samples were collected then microcystin-LR was immediately added to the cultures. Samples were collected again at 15, 30, 45, and 60 min. The samples for RNA extraction were immediately added to 25 mL of ice and centrifuged at 10,000 x g for 7.5 minutes at 4°C. The cell pellet was placed in -80°C freezer where it was stored until RNA extraction. Lipid samples were collected on 0.45-μm polycarbonate filters and flash frozen in liquid nitrogen. They were stored at -80°C until extraction. For metabolite samples, cultures were established as described except 120 mL master cultures were grown in 500 mL flasks and were divided into 30 mL treatment cultures grown in 150 mL flasks. Samples were collected and handled as described for lipid samples. Data from the six replicates (three RNA-seq lipid experiments and three metabolite experiments) were used in growth rate calculations.

### RNA extraction and sequencing

Total RNA was extracted from samples using the hot phenol method described in Wen *et al*. [32]. Genomic DNA was removed with the Turbo DNA-*free* Kit (Ambion, Life Technologies) using a modified protocol. After total RNA extraction, approximately 40 μg of total RNA was suspended in 85 μL of RNase-free water. 10 μL of 10x buffer and 4 μL of Turbo DNase enzyme were added. The solution was incubated at 37°C for 40 min, extracted once each with phenol:chloroform and chloroform, then ethanol precipitated. Samples were considered DNA-free if no bands were visible in an agarose gel after 30 cycles of PCR amplification targeting the 16S rRNA gene using primers 27F and 1522R. Ribosomal RNA was depleted using the MICROBExpress Kit (Ambion, Life Technologies) following manufacturer’s instructions.

RNA samples were sent to the Molecular Resource Center, University of Tennessee Health Science Center (Memphis, TN) for library preparation and sequencing. Forty-five libraries were prepared (one for each treatment/time point/replicate combination) and sequenced to a targeted depth of ~50-fold. Barcoded libraries were prepared using the Ion Total RNA-Seq Kit for AB Library Builder System (Life Technologies). Single-end reads from pooled libraries were sequenced on the Ion Torrent Proton Sequencer using the Proton I chip (Life Technologies). Sequence information has been uploaded to the NCBI Sequence Read Archive under project number PRJNA349165.

### Bioinformatic analysis

Sequencing reads were mapped to gene regions of the *E*. *coli* MG1655 reference genome (GenBank U00096.3) using CLC Genomics Workbench (ver 8.5.1) with default parameters for mismatch, insertion, and deletion costs and custom settings of 0.6 for length fraction and 0.85 for similarity fraction. Expression values for each gene were calculated from unique gene reads and normalized by library size yielding the expression value of total counts per million (TCPM). Raw mapped read counts were exported from CLC and used as input for the DESeq2 software program, which were used to identify genes differentially expressed between treatment and control [33, 34]. Default parameters were used in the DESeq2 analysis. P-values of individual genes were adjusted for false discovery rate (FDR) [35]. Genes with an FDR-adjusted p-value < 0.1 and whose expression value in the treatment was either 1.5x higher or lower (fold change) than that in control were considered significantly differentially expressed. Genes were annotated following EcoGene 3.0 [36] and supplemented with information from EcoCyc [37].

### Metabolite extraction

Metabolite extraction was performed at 4°C unless otherwise specified. Frozen filters (0.45-μm pore-size, polycarbonate) were placed in petri dishes and unfolded into 1.3 mL extraction solvent consisting of a 40:40:20 mixture of HPLC grade methanol, acetonitrile, water with 0.1 M formic acid [38]. Extraction proceeded at -20°C for 20 min. Filters were then flipped and the extraction solvent was pipetted over filters to wash the cells free of the filters. Extraction solvent was then transferred to a 1.5 mL microcentrifuge tube. An additional 400 μL of extraction solvent was used to further wash cells from the filter. The additional solvent was transferred to the same microcentrifuge tube. The samples were centrifuged at 16,100 x g for 5 min. The supernatant was transferred to new vials and the cell pellet was resuspended in 200 μL of extraction solvent. The extraction was allowed to proceed for another 20 min at -20°C. Samples were again centrifuged at 16,100 x g for 5 min. The supernatants were transferred to the same set of vials that were then dried under a stream of N_2_. Sterile water (300 μL) was added to resuspend solid residue and transferred to 300 μL autosampler vials.

### Lipid extraction

Filters were extracted for 15 min in 800 μL of extraction solvent consisting of a 15:15:5:1:0.18 mixture of 95% ethanol, water, diethylether, pyridine, and 4.2N NH_4_OH. The solvent was transferred to ~100 μL of glass beads, vortexed and placed in a 60°C water bath for 20 minutes. The beads were centrifuged at 10,000x g for 10 min and the supernatant transferred to a clean glass vial. A second extraction using 800 μL of fresh solvent was repeated as described. The residual glass beads were washed by re-suspending in 300 μL of water-saturated 1-butanol and 150 μL of water, vortexing, then centrifuging at 10,000 x g for 2 min. The top organic layer was transferred to a glass vial containing the solvent from the two previous extractions. The beads were washed a second time as described. The samples were dried under a stream of N_2_ and re-suspended in 300 μL of a 9:1 mixture of methanol and chloroform.

### Metabolite UPLC–MS analysis

Samples were loaded into an Ultimate 3000 autosampler (Dionex, Sunnyvale, CA) kept at 4°C. A 10-μL sample was injected through a Synergi 2.5 micron Hydro-RP 100, 100 x 2.00 mm LC column (Phenomenex, Torrance, CA, USA) with column compartment maintained at 25°C. The mass spectrometer was run in fullscan in negative ionization mode using a method adapted from Lu *et al*. [39]. Samples were ionized via an electrospray ionization (ESI) source through a 0.1 mm internal diameter fused silica capillary tube before analysis on an Exactive Plus orbitrap mass spectrometer (Thermo Scientific, San Jose, CA). A spray voltage of 3 kV was used with the nitrogen sheath gas set to a flow rate of 10 units and a capillary temperature of 320°C. The automated gain control target was set to 3 x 10^6^, with a resolution of 140,000 and a scan window of 85 to 800 m/z for 0 to 9 min and 110 to 1000 m/z from 9 to 25 min. Solvent A consisted of 97:3 HPLC grade water: methanol, 10 mM tributylamine, and 15 mM acetic acid. Solvent B was HPLC grade methanol. The gradient from 0 to 5 min was 0% B, from 5 to 13 minutes was 20% B, from 13 to 15.5 min was 55% B, from 15.5 to 19 min was 95% B, and from 19 to 25 min was 0% B with a flow rate of 200 μL/min.

### Lipid UPLC–MS analysis

Samples were loaded into an Ultimate 3000 autosampler (Dionex, Sunnyvale, CA) kept at 4°C and a 10-μL aliquot was injected through a Kinetex HILIC column (150 mm x 2.1 mm, 2.6 μm) (Phenomenex, Torrance, CA, USA). The eluent was analyzed by an Exactive Plus Orbitrap mass spectrometer (Thermo Fisher Scientific, San Jose, CA) equipped with an ESI probe. Chromatography ran for a total of 35 min with mobile phase A and B consisting of 10 mM aqueous ammonium formate pH 3 and 10 mM ammonium formate pH 3 in 93% (v/v) ACN, respectively. The beginning of the gradient was 100% B for 1 min; 81% B from 1 to 15 min, 48% B from 15 to 15.1 min, maintained at 48% B from 15.1 to 25 min, increased to 100% B from 25 to 25.1 min, and finally re-equilibrated at 100% B from 25.1 to 35 min. A flowrate of 0.2 mL/min was maintained throughout the separation. The column oven temperature was kept at 25°C. The spray voltage was set to 4 kV with a heated capillary temperature of 350°C. The sheath gas flow was set to 25 units and the auxiliary gas set to 10 units. The MS used a resolution of 140,000 with a scan range of 100–1500 m/z for full-scan mode and a scan range of 100–1500 m/z for all ion fragmentation scans. The normalized collision energy was 30eV with a stepped collision energy of 50%. Each sample was run in positive and negative mode. Lipid classes were identified by their fragments using Xcalibur software (Thermo Fisher Scientific, San Jose, CA).

### Metabolite and lipid data analysis

Raw files created by Xcalibur were converted to open source mzML format [40] using the ProteoWizard software [41]. MAVEN software [42] was used to perform nonlinear retention time correction for each sample and known metabolite and lipids were manually selected based on retention time and exact mass [42, 43]. Tests for differences of individual metabolites and lipids were performed using ANOVA implemented in the R language. Multivariate analysis was performed using Primer7 version 7.0.9 [44]. Euclidian distances were used in constructing the similarity table after the data were 4^th^ root transformed.

## Results

### Effects of microcystin-LR on growth rate

Average growth rate in the 1-mg/L and 10-mg/L treatments was μ = 0.46/hr (generation time = 1.51 hr) and in control was μ = 0.47/hr (generation time = 1.47 hr) and did not statistically vary (ANOVA p-value = 0.91). Thus, exposure to MCLR at concentrations of 1 mg/L or 10 mg/L had no effect on growth rate. Optical densities of cultures at the beginning and end of the experiment are illustrated in S1 Fig. A 12-hr growth curve representing conditions of master cultures is provided in S2 Fig.

### RNA sequencing output and quality control

Sequencing on the Ion Torrent Proton Sequencer generated a total of 95,534,894 single-end reads from 45 libraries resulting in an average library size of 2,122,998 reads (SE, ± 68,511 reads). Average length of reads was 146 bp and average phred score was 24.2. Reads with a phred score less than 17 were removed during post-processing and are not included in the completed libraries and are not reflected in the reported totals.

Reads were mapped to the *E*. *coli* reference genome using the RNA-seq Analysis package of CLC Genomics Workbench. Of the total reads, 77,183,360 (80.8%) mapped to ribosomal RNA genes and were removed from libraries, leaving 18,351,534 non-ribosomal reads (19.2% of total) for further analysis. A total of 13,184,412 reads mapped to protein-encoding genes and 126,141 mapped uniquely to tRNA genes. Thus each library had on average 295,790 mapped reads upon which calculations of gene expression were based. Uniquely mapped reads per library ranged from a high of 614,653 to a low of 95,653. The average depth of sequencing calculated using total reads was ~67 fold; the average depth of sequencing calculated using reads mapped to protein-encoding genes was ~9 fold. A summary of reads is provided in S1 Table.

Consistency in gene expression between each pair of biological replicates was quantified by calculating Pearson’s correlation using genes with non-zero counts in at least one replicate [45]. The mean correlation between all replicate pairs was *r* = 0.98. Similar correlations have been viewed as a mark of reliability in benchmarking RNA-seq studies [45–47].

**Fig 1:**
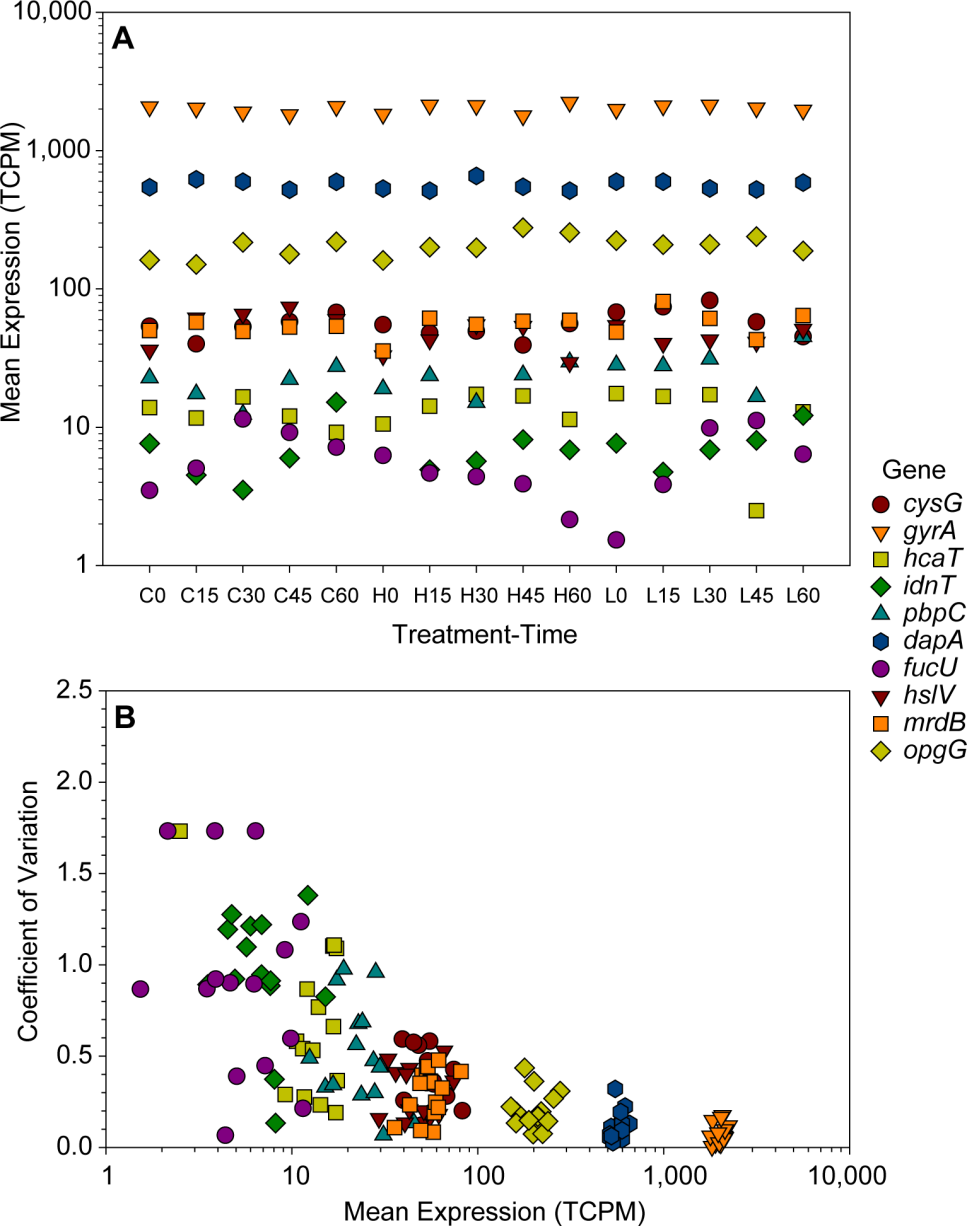
Expression of reference genes. (A) Mean expression in total counts per million (TCPM) of 10 reference genes for each treatment/time point combination. The X-axis labels indicate the treatment and time point: “C” = control, “H” = 10 mg/L treatment, and “L” = 1 mg/L treatment. Expression values are the mean of three replicates. (B) Mean expression vs. coefficient of variation for 10 reference genes. Each point represents the mean expression of three replicates and the corresponding coefficient of variation for a treatment/time point combination. Gene abbreviations: *cysG* (siroheme synthase), *gyrA* (DNA gyrase, subunit A), *hcaT* (3-phenylpropionate transporter), *idnT* (L-Idonate transporter), *pbpC* (peptidoglycan glycosyltransferase), *dapA* (dihydrodipicolinate synthase), *fucU* (L-fucose mutarotase), *hslV* (heat-inducible protease subunit), *mrdB* (shape, elongation, division and sporulation family protein B), *opgG* (osmoregulated periplasmic glucans biosynthesis protein G).

To empirically determine valid levels of read-number significance, the expression of 10 canonical reference genes (reported in the literature to be stably expressed in E. coli [48, 49]) was examined. Fig 1A illustrates the mean expression in TCPM for each reference gene at each treatment-time combination. Average TCPM ranged from a low of ~6 to a high of ~2,000. Seven reference genes (*cysG*, *gyrA*, *pbpC*, *dapA*, *hslV*, *mrdB*, *opgG*) had a mean TCPM greater than ~24 and displayed mean coefficients of variation < ~0.5 (Fig 1B). These results further validate the RNA-seq data and verify reliability of gene expression estimates derived therefrom. Three reference genes (*hcaT*, *idnT*, *fucU*) had a mean TCPM < 15 and had high coefficients of variation (CV > ~0.7), suggesting that expression estimates at or below these levels may be unstable. This is a recognized phenomenon and is managed in DESeq2 by shrinking estimates of fold change for weakly expressed genes [33]. In our study, of the 4,498 annotated features in the *E*. *coli* MG1655 genome, ~310 had no detectable transcripts in any treatment and ~2,660 had expression > 15 TCPM, providing robust statistical power to detect differentially expressed genes over ~66% of the genome.

### Analysis of differential gene expression

Genes differentially expressed in microcystin treatments relative to control were calculated for each time point independently. DESeq2 identified a total of only nine genes as significantly differentially expressed between both the 1-mg/L and 10-mg/L treatments (Table 1). Two genes (*hisR*, *serV*) were differentially expressed in both the 1-mg/L and 10-mg/L treatments with the direction and magnitude of the fold change in close agreement between treatments. The remaining seven genes were differentially expressed in the 10-mg/L treatment only. Of these, one gene (*infC*) was up-regulated while all others were down-regulated. A detailed description of all differentially expressed genes by treatment, time point, and functional category is provided in Table 1. Surprisingly, 8 out of 9 differentially expressed genes coded for RNAs or proteins involved in RNA processing and all were down regulated: five coded for tRNAs, two coded for RNAs involved in translation, and one coded for a protein subunit of RNase P involved in RNA processing. Criteria used to identify significantly differentially expressed genes (fold change ≥ 1.5, *p* ≤ 0.1) were deliberately non-stringent to prevent excluding potentially important telltale genes that might fail tighter stringency tests. To confirm the unexpectedly low number of significant genes, the data were reprocessed using the edgeR algorithm [34]. There was no notable difference in results between the two methods.

**Table 1:** Description of differentially expressed genes by time point and treatment. FDR–False Discovery Rate corrected.

	Treatment	Time		Fold	FDR	Functional	
Algorithm	(mg/L)	Point	Gene	Change	p-value	Category	Protein Description
DESeq2	10	45	*ffs*	-2.1	0.0003	cotranslational export	4.5S RNA component of Signal Recognition Particle (SRP)
DESeq2	10	45	*glyT*	-2.2	0.0001	tRNA	glycine tRNA(UCC) 2, UGA suppression
DESeq2	1, 10	45, 45	*hisR*	-1.9, -2.0	0.0003	tRNA	histidine tRNA(GUG)
DESeq2	10	45	*rnpB*	-1.9	0.0001	RNA modification	subunit of RNase P; involved in tRNA and 4.5S RNA-processing
DESeq2	10	45	*selC*	-1.8	0.0085	tRNA	selenocysteyl tRNA(UCA) (converted from serine tRNA)
DESeq2	1, 10	30, 45	*serV*	-1.5, -1.9	0.0012	tRNA	serine tRNA(GCU) 3
DESeq2	10	45	*ssrA*	-1.6	0.0003	trans-translation	tmRNA; tRNA-Ala and mRNA, tags proteins for degradation
DESeq2	10	45	*tyrU*	-1.8	0.0018	tRNA	tyrosine tRNA(GUA) 2
DESeq2	10	60	*infC*	1.5	0.0773	translation	translation initiation factor IF-3; unusual AUU start codon

### Expression of oxidative stress and cell envelope stress genes

Previous studies have reported that exposure to MCRR or MCLR produced oxidative stress and cell envelope stress in bacteria and/or cyanobacteria [27, 50, 51]. In our study, no genes associated with oxidative or cell envelope stress were differentially expressed due to MCLR exposure. To examine patterns of stress-related genes in greater detail, mean expression of genes from well-characterized stress response pathways were compared for each time point. Genes selected for this comparison have been characterized in at least two independent studies as being strongly and positively regulated in response to either oxidative or cell envelope stress. Our rationale was that these criteria would provide the best opportunity for identifying genes with expression altered due to treatment, but whose shift in expression failed to be detected by DESeq2 due to statistical cut-off limits.

**Fig 2:**
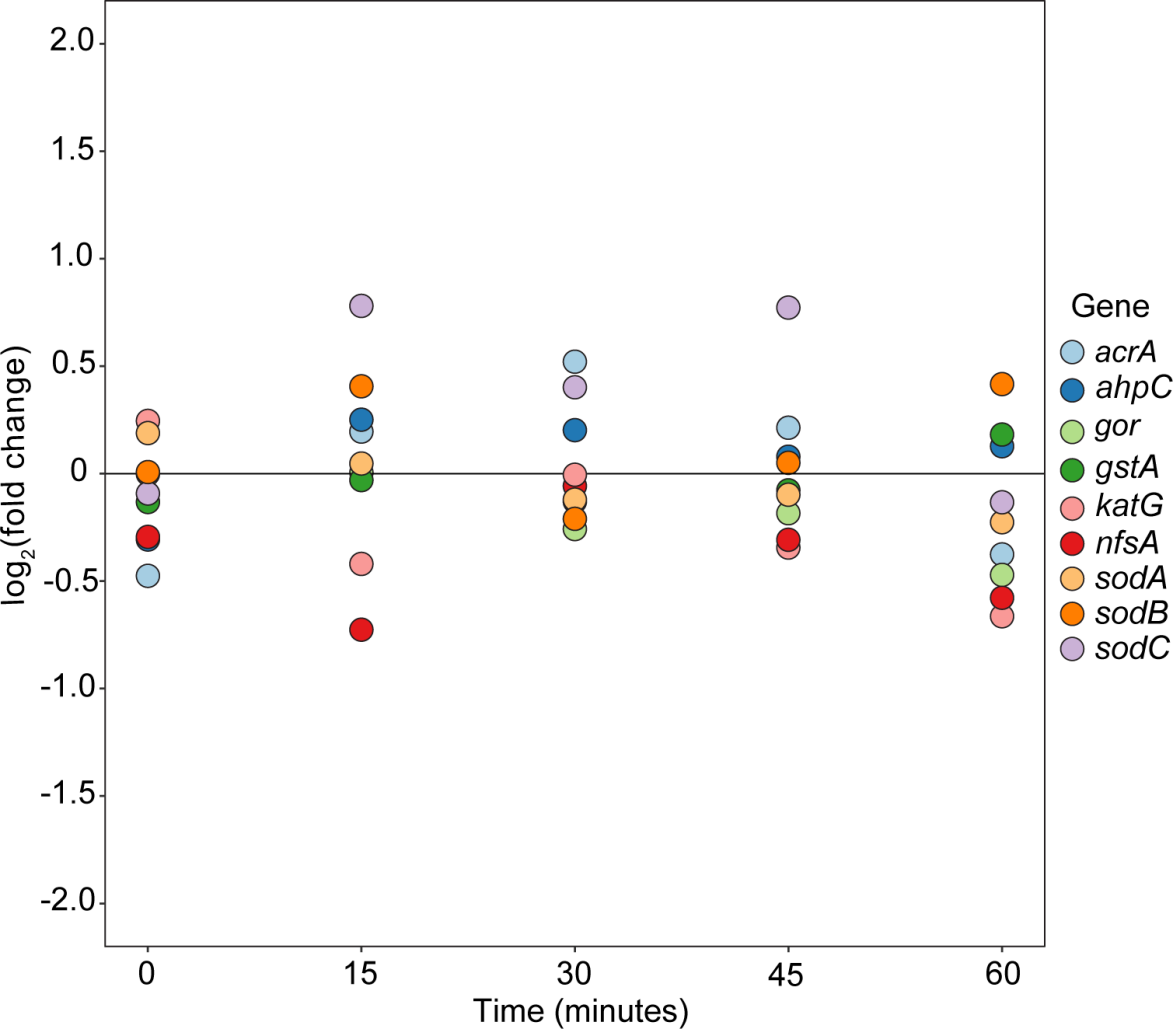
Fold change in oxidative stress gene expression for 10-mg/L MCLR treatment. Each point represents the log_2_ fold change relative to control at a given time point. The horizontal line at 0 represents equal expression in treatment and control. Gene abbreviations: *acrA* (multidrug efflux pump membrane fusion protein), *ahpC* (alkyl hydroperoxide reductase), *gor* (glutathione reductase), *gstA* (glutathione *S*-transferase), *katG* (catalase), *nfsA* (Nitroreductase A), *sodA* (Mn-containing superoxide dismutase), *sodB* (Fe-containing superoxide dismutase), *sodC* (Cu-Zn-containing superoxide dismutase).

Fold changes relative to control for 9 key oxidative stress response genes from the 10-mg/L treatments are shown in Fig 2. Importantly, each of these genes had expression > 24 TCPM at each time point. Fold-changes are distributed almost symmetrically around and fall near the “zero-line” for all time points, suggesting that expression of these genes did not vary from control due to treatment. Taken together, these patterns further suggest that in our study, MCLR failed to generate detectable oxidative stress in *E*. *coli*.

**Fig 3:**
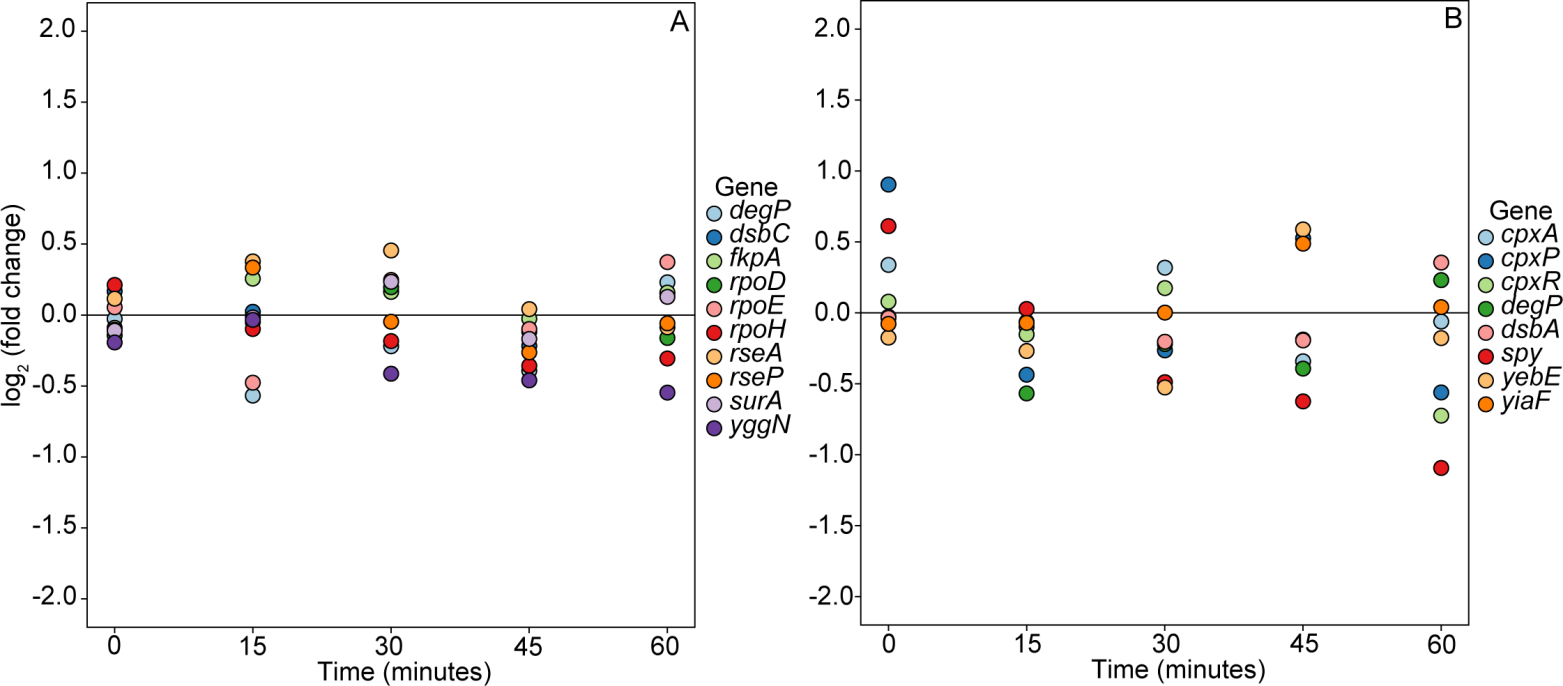
Fold change in envelope stress gene expression for 10-mg/L MCLR treatment. (A) Genes of the σ^E^ regulon. (B) Genes of the Cpx regulon. Each point represents the log_2_ fold change relative to control at a given time point. The horizontal line at 0 represents equal expression in treatment and control. Gene abbreviations: *degP* (periplasmic serine endoprotease), *dsbC* (disulfide bond isomerase), *fkpA* (peptidyl-prolyl *cis-trans* isomerase), *rpoD* (RNA polymerase subunit, sigma 70), *rpoE* (RNA polymerase, sigma E), *rpoH* (RNA polymerase, sigma H), *rseA* (anti-RpoE sigma factor), *rseP* (intramembrane zinc metalloprotease), *surA* (peptidyl-prolyl *cis-trans* isomerase), *yggN* (DUF2884 family periplasmic protein), *cpxA* (sensory histidine kinase), *cpxP* (periplasmic adaptor protein), *cpxR* (DNA-binding transcriptional dual regulator), *dsbA* (protein disulfide oxidoreductase), *spy* (ATP-independent periplasmic chaperone), *yebE* (DUF533 family inner membrane protein), *yiaF* (DUF3053 domain-containing protein).

*E*. *coli* has five well characterized envelope stress signaling pathways [52, 53]. Fold change relative to control of genes from two of these pathways, the σ^E^ regulon and the Cpx pathway, are shown in Fig 3A and 3B, respectively, for the 10-mg/L treatment. Fold-changes are distributed tightly around the zero-line. Genes from the Bae, Psp, and Rcs pathways showed similar trends with slightly more variation (S3 Fig). Each of the genes included in Fig 3 and S1 Fig had expression > 24 TCPM. Together, fold-change patterns in these signaling pathways provide no evidence that cells were experiencing MCLR-induced envelope stress.

Ethanol was used as a solvent to reduce loss of microcystin due to adsorption onto plastic surfaces during preparation and handling [54]. Addition of microcystin to treatment cultures resulted in a final ethanol concentration of 1% (v/v); an equivalent amount of ethanol was added to control. The possibility exists that induction of genes in stress pathways due to ethanol could mask responses due to microcystin, reducing our ability to detect real responses. To address this concern, we analyzed 30 genes for differential expression at time 15 min vs. time 0 and at time 30 min vs. time 0 in the control treatment. Samples at time 0 min were taken immediately prior to addition of ethanol as solvent control, so this analysis gives an indication of the effects of ethanol. We used the same oxidative stress (9 genes) and envelope stress (21) marker genes featured in Figs 2 and 3 and S1 Fig. Five genes out of these 30 were differentially expressed, but only three had increased expression relative to 0 min. *cpxP* was up-regulated ~2.5 fold at 15 and 30 min, *pspA* was up-regulated 2.2 fold at 15 min, and *degP* was up-regulated 1.5 fold at 30 min.

### Analysis of metabolite pools and lipids

Samples to analyze small molecules were taken from cultures distinct from those from which RNA/lipid samples were taken: the experimental design and sampling methods were identical to those used for the RNA/lipid cultures. A total of 87 metabolites were detected from a standard screening panel of ~ 270 compounds. Each metabolite was analyzed independently at each time point to test for differences due to treatment. No individual metabolite was found to be significantly different at any time point after FDR adjustment for multiple comparisons.

Several metabolites have been identified in benchmarking studies as useful biomarkers of bona fide oxidative stress [55, 56]. Within 20 min of stress-inducing treatments, relative concentrations of alanine and asparagine increase, while that of methionine decreases [56]. This is accompanied by pronounced decreases in glucose-6-phosphate, 3-phosphoglycerate, malic acid, and 2-oxoglutarate, metabolites associated with glycolysis and the TCA cycle [56]. Additionally, detectable shifts of glutathione (GSH) and glutathione disulfide (GSSG) occur within 30 min of induced stress [55]. To screen for the possible effects of microcystin-induced oxidative stress, we examined the pattern of these metabolites over the time series. Though variable, alanine, asparagine, and methionine increased relative to control at 30 min in the 10-mg/L treatment (S4 Fig). The pattern of alanine and asparagine were consistent with oxidative stress, but that of methionine was not. None of these changes were significant. Glucose-6-phosphate, 3-phosphoglycerate, malic acid, 2-oxogluatrate, GSH, and GSSG showed patterns similar to those of the control (S5 Fig and S6 Fig). Overall, MCLR appeared to cause little change in metabolite pools. No significant differences in lipid composition were found between treatments. Metabolite and lipid data normalized to optical density is provided in S1 and S2 Files.

## Discussion

The effect of microcystin on eukaryotic cells has been studied extensively (reviewed in [3] and [57]). However, its effect on heterotrophic bacteria, which are the numerically dominant members of environments inhabited by microcystin producers, has received surprisingly little attention. Previous studies have reported that microcystin-RR generates oxidative and cell envelope stress in *E*. *coli* [26, 27, 58]. Based on these reports, we hypothesized that exposure to microcystin would trigger differential expression in genes that respond to oxidative and cell envelope stress and that metabolite concentrations would alter as cells respond to induced stress. To test our hypotheses, we exposed *E*. *coli* to 1 and 10 mg/L microcystin-LR and monitored cellular responses via gene expression and shifts in metabolite pools. *E*. *coli* was selected for its position as a model heterotrophic bacterium and for the wealth of tools and data available on this species for comparative study. It was used in previous studies demonstrating a stress phenotype and thus allowed us to test reproducibility while using different experimental methods in monitoring responses. Additionally, *E*. *coli* has some ecologic relevance as it is widely distributed in freshwater systems [59] and strains of non-fecal origin have been found associated with large *Microcystis* blooms in highly eutrophic freshwater lakes [60]. Overall, it was hoped that responses of *E*. *coli* could serve as a model from which responses in related heterotrophs might be better understood and predicted.

Exposure to either 1 or 10 mg/L MCLR had no effect on growth of *E*. *coli* over the course of 1 h. On this topic, reports in the literature are inconsistent. We found no studies in the literature that tested MCLR and used methods similar enough to ours to allow direct comparisons. However, two studies investigated effects of MCLR on *E*. *coli* grown on agar and reported conflicting results. In the first, there was no inhibition of growth from either MCLR or MCRR at concentrations of up to 8 mg/L when grown on Merck Test agar [22]. In the second, Valdor *et al*. [24] found that growth on pancreatic peptone agar was inhibited by MCLR at concentrations greater than 5 mg/L and by MCRR at concentrations greater than 12.5 mg/L [24]. Thus, in the Valdor study, a direct comparison of MCLR and MCRR showed that MCLR was more inhibitory. However, in a comparison of our work *vs*. that of Yang *et al*. [27], MCRR seemed to be more inhibitory. Yang *et al*. reported that in nutrient broth, ≥ 10 mg/L MCRR produced a significant growth reduction in *E*. *coli*. In our study, 10 mg/L of MCLR had no effect on *E*. *coli* growth in minimal media + glucose. It is possible that the difference in results between Yang *et al*. and our study could be explained by use of different media. Additionally, it seems that the effect may be congener specific and sensitive to growth conditions.

RNA-seq has been used to monitor the response of oxidative-stress-induced *E*. *coli* in a number of studies, with detectable shifts in expression of key oxidative stress genes occurring within 20–30 minutes of inducing conditions [61, 62]. In our study, gene expression data monitored for a period of one hour post-induction provide no evidence that exposure to MCLR at 1 or 10 mg/L triggered oxidative stress in *E*. *coli*. Here again, our results stand in contrast to previously published research. A key study showed that *E*. *coli* exposed to 10 or 15 mg/L of MCRR produced dose-dependent increases in intracellular ROS and glutathione concentrations and increased activities of catalase, superoxide dismutase, and glutathione reductase, all within 30 min of exposure [27]. At 60 min in 10 mg/L MCRR, ROS concentrations had increased ~3 fold over control and responses of other parameters increased between ~2–3.5 fold. Peak concentration or activity occurred at 60 min post-exposure for all measured parameters. Even the reduced concentration of 5 mg/L MCRR produced significant increases in ROS concentration and superoxide dismutase and catalase activities at the same time scale.

At first glance, the different results between our experiment and prior studies are explainable by the use of different congeners. However, work investigating the effects of microcystin on cyanobacterial species complicates this interpretation. Using *Synechococcus elongatus*, Hu *et al*. [50] showed that exposure to 0.1 mg/L of MCRR produced a 3-fold increase in ROS concentrations and increases in glutathione S transferase and glutathione peroxidase activity. In a separate study using *Synechocystis* PCC6803, exposure to exposure to 1 and 5 mg/L MCRR triggered increases in the oxidative stress genes *gpx1* (glutathione peroxidase, ~12-fold), *katG* (catalase, ~4-fold), and *sodB* (Fe superoxide dismutase, ~5-fold) [63]. These studies along with Yang *et al*. [27], suggest that MCRR generates oxidative stress in diverse bacterial cells. Additionally, a study by Vassilakaki and Pflugmacher [51], also using *Synechocystis* PCC6803 showed that the congener MCLR at concentrations as low as 1 μg/L produced 2-fold increases in intracellular hydrogen peroxide concentration and increases in activities of superoxide dismutase (~2-fold), catalase (~5-fold), glutathione S transferase (~4-fold), and glutathione reductase (~3.5-fold). Thus it has been shown that in cyanobacteria, both MCLR and MCRR produce oxidative stress, and that in *E*. *coli*, MCRR produces oxidative stress.

No mechanism for microcystin-induced generation of oxidative stress in bacterial cells has been proposed. But if the mechanism is the same in cyanobacterial and heterotrophic cells, then based on previous findings, we expected that MCLR would produce oxidative stress in *E*. *coli*. It is possible that the mechanism at work in cyanobacterial cells is different from that in heterotrophic cells, leaving the LR congener inactive in *E*. *coli*. Another explanation for apparent lack of oxidative stress in our study is that the response was not strong enough to reveal itself in the form of induced gene expression. However, in *Synechocystis* PCC6803, MCLR produced 2–5 fold increases in either peroxide concentrations or oxidative stress enzyme activities [51]. The oxidative stress response in *E*. *coli* is well characterized [64], and it seems unlikely that a stress of similar magnitude would go undetected in the expression of regulatory pathways. Moreover, the sequencing depth of our RNA-seq data was adequate to establish reliable gene expression estimates, as shown by reference gene patterns, and to detect significant shifts in gene expression [47]. Thus it would seem that congeners do not explain the differences observed, or that a different mechanism is at work in *E*. *coli* vs. cyanobacteria. The effects of different media and experimental conditions might also contribute.

To contend with a possible masking effect due to use of ethanol as a microcystin solvent, gene expression data were analyzed to determine if there was differential expression of oxidative stress genes in the control at time 15 min vs. time 0 and in the control at time 30 min vs. time 0. Results of this analysis must be interpreted with some caution, because it is not possible to identify whether potentially differentially expressed genes are due to effects of ethanol or to the passage of time during batch culture. But, the analysis does give a solid indication of whether these genes were in fact differentially expressed relative to time 0. *sodB*, which is typically up-regulated during oxidative stress, was the only gene identified in this analysis, and it was down-regulated at time 30 min, suggesting an effect due to ethanol, if any, was negligible.

Monitoring of metabolites that are altered by oxidative stress provided no clear evidence that such stress was induced. While alanine and asparagine accumulated in the 10-mg/L treatment, consistent with oxidative stress, other biomarkers, including GSH, GSSG, and metabolites from glycolysis and the TCA cycle were consistent with the control. In the case of alanine and asparagine, the departures from control were not significant, forcing us to conclude that metabolomics profiles were not altered due to treatment. Thus, the collective data are consistent with the interpretations that generation of oxidative stress in *E*. *coli* by microcystin is congener specific and that MCLR fails to generate oxidative stress under the conditions tested. A corollary is that MCRR apparently generates oxidative stress in *E*. *coli* by a mechanism not activated by MCLR.

*E*. *coli* has five characterized signaling pathways whose functions are to sense various perturbations to the cell envelope and respond by up-regulating expression of genes that encode proteins needed to repair damage [53, 65]. A frequent inducing cue for the pathways are misfolded periplasmic or outer membrane proteins. However, the pathways respond to diverse stimuli including the integrity of the outer membrane [66–68]. An earlier study reported that MCRR permeabilized the outer membrane of *E*. *coli* and caused leakage of periplasmic proteins, as measured using a chromogenic β-lactamase assay [26]. We predicted that if MCLR functioned in a manner similar to MCRR, increased expression in genes from one or more of the response pathway regulons would be observed. However, we observed no evidence from gene expression suggesting MCLR triggered envelope stress in *E*. *coli*. MCRR is structurally similar to polymyxin B nonapeptide (PMBN), and it is proposed that MCRR generates envelope stress in Gram-negative bacteria by a mechanism similar to that of PMBN [26, 69, 70]. The five positively charged free amino groups of PMBN interact with the anionic groups of LPS, disrupting the quasicrystalline structure of the outer leaflet of the outer membrane, producing the observed increased permeability [69]. MCRR has two positively charged amino groups and a net charge of 0, while MCLR has one positively charged amino group and a net charge of -1. These differences offer a possible explanation for their differing ability to generate outer membrane stress.

A possible masking effect of ethanol was of greater concern in monitoring cell envelope stress than in oxidative stress. This concern was addressed in the same manner as for oxidative stress. Differential expression analyses in the control identified three genes (*cpxP*, *pspA*, *degP*) that were significantly up-regulated among the 21 envelope stress marker genes monitored. However, the response in our study was weak and incomplete. In comparison, Bury-Moné et al. [52] observed ~3.5 fold and >5 fold induction of *cpxP* in treatments of 3% and 5% (v/v) ethanol, respectively. *cpxP* is reported as the most sensitive and highly induced gene of the regulon, with expression increasing ~15 fold upon induced membrane stress [71]. *pspA* is strongly induced upon treatment with ethanol in a dose-dependent manner [72, 73]. In our experiment, *cpxP* and *pspA* were weakly induced. *degP* was very weakly induced and just cleared the criterion (1.5x fold change) used to designate differentially expressed genes. Thus, while the masking effect can’t be entirely ruled out, any effect appears slight and incomplete and not enough to obscure a response to envelope damage. Although this makes us more cautious, it doesn’t alter our conclusion that MCLR does not generate significant envelope stress under our experimental conditions.

A total of 9 genes were differentially expressed as determined by DESeq2. Products of these genes show a high degree of association in function: five are tRNAs and four are involved in RNA processing/stabilization. All but one were down-regulated. The biological meaning of this isn’t clear, although tRNAs and their fragments have recently been shown to play regulatory roles in both eukaryotes and prokaryotes (reviewed in [74]). tRNA gene regulation is growth rate dependent and under stringent control, yet growth rate among treatments was nearly identical, and only 5 tRNAs of 86 total in the *E*. *coli* genome [75] were differentially expressed. Thus, an explanation of this pattern is puzzling. In other studies, interpretation of tRNA expression has been inconclusive and sometimes without significance [74].

Heterotrophic bacteria co-occur with microcystin-producing cyanobacteria and are thus potentially exposed to microcystin for extensive periods. Our experiment tested the effects of exposure to MCLR over a 1-hr period during exponential growth and found little to no evidence of effect. The question then arises about whether our experimental methods could capture the full response of *E*. *coli* to exposure to microcystin. Our design was based, in part, on the fact that regulatory pathways that respond to oxidative and envelope stress are well characterized in *E*. *coli* [64, 68] and are known to respond rapidly to bona fide stress stimuli. In comprehensive benchmarking studies using *E*. *coli*, measurable responses to oxidative stress occurred within 10 min of stress induction [56]. In fact, over a time series, the largest number of changes in both gene expression and metabolite concentrations occurred within 10 min, with ~200 genes being differentially expressed. Key oxidative response genes, like *katG*, were up-regulated ~40-fold [56]. A number of studies have shown that response to oxidative stress is rapid with measurable responses occurring within 20–30 min [55, 61, 62]. Likewise, the envelope stress response systems are known to respond rapidly. Measureable responses in the σ^E^ system can occur within 3 min of initiating envelope stress [76], while changes greater than 50-fold have been measured in genes of the Rcs system within 40 min of initiating stress [66].

Our experimental design was based in part on previous reports of microcystin-induced stress in *E*. *coli*. Yang et al. [27] reported significant increases in oxidative-stress-enzyme activities in *E*. *coli* within 30 min post-exposure to MCRR. In similar time frame, Dixon et al. [26] reported measurable amounts of periplasmic proteins being spilled from *E*. *coli* within 20 min post-exposure to MCRR due to permeabilisation of the outer membrane. In these latter examples, a congener of microcystin produced measurable effects in a time frame consistent with our experimental design and consistent with the idea that response to stress occurs within minutes. It is possible in our case that longer exposure to MCLR might have produced a measurable effect. But in batch culture, longer exposure times would have meant measuring the response of cells in late exponential phase or early stationary phase when cells are known to be more resistant to a variety of stresses, especially oxidative stress [77]. Thus it is unlikely that longer exposure times would have produced different results. Given our experimental design, we cannot draw conclusions about longer exposure times. However, we can conclude that we saw no evidence of a stress response to MCLR within a time frame used by other researchers to show responses to the congener MCRR.

## Conclusions

A goal of this study was to improve understanding of how microcystin affects the physiology of heterotrophic bacteria within the context of a confounding body of research. The literature addressing this topic is small and contradictory, but suggest that the effect is species, congener, and growth-condition-dependent. Data presented here test the most abundant microcystin congener using methods that monitor global cellular responses. Our results show that MCLR has little effect on the physiology of *E*. *coli* MG1655 under the tested conditions. Given previous work, our findings point to a potential difference in mechanism through which MCLR interacts with heterotrophic bacteria vs. cyanobacteria such as *Synechocystis* spp. This work suggests that even very high concentrations of MCLR have little influence on the physiology of *E*. *coli*. Limited by the extent to which results from *E*. *coli* are transferable to a broader group of bacteria, our findings suggest MCLR has limited potential to alter the physiology or ecology of a segment of heterotrophic bacteria that co-occur with toxic cyanobacterial blooms. This provides additional support for the growing consensus that microcystin is produced by cyanobacteria to influence one or more internal metabolic or physiological process. Ultimately, understanding the reason cells produce these compounds is an important step towards our ability to constrain their production in natural systems.

## Supporting information

S1 Table Summary of RNA sequencing libraries. (DOCX)

S1 Fig O.D._600_ of treatment and control cultures. O.D._600_ measurements of cultures at the start (time = 0) and end (time = 60 min) of treatments. Symbols represent actual data points of biological replicates. The horizontal bars represent the mean.
(TIF)

S2 Fig Example growth curve of *E*. *coli*. Growth curves of *E*. *coli* in M9 minimal medium with addition of 4 g L^-1^ glucose and 1 mg L^-1^ thiamine HCl. These curves were generated in preliminary experiments and represent conditions identical to those of the master cultures described in Methods. Treatments were imposed on cultures approximately 8 hr after inoculation when O.D._600_ was ~0.3. Error bars represent 1 S.E., but are smaller than symbol size. Growth rates calculated from these curves were identical (μ = 0.47 hr^-1^) to those calculated from the control cultures during the experiment. n = 2.
(TIF)

S3 Fig Fold change in Bae, Psp, and Rcs regulon gene expression for 10-mg/L MCLR treatment. Each point represents the log_2_ fold change relative to control at a given time point. The horizontal line at 0 represents equal expression in treatment and control. Gene abbreviations: Bae regulon: *mdtB (*multidrug efflux pump RND permease subunit). Psp regulon: *pspA (*phage shock protein A). Rcs regulon: *osmB (*osmotically and stress inducible lipoprotein), *osmY (osmotically inducible* periplasmic chaperone), *surA (*periplasmic OM porin chaperone).
(TIF)

S4 Fig Relative concentration of amino acids responsive to oxidative stress. Error bars represent 1 S.E.
(TIF)

S5 Fig Relative concentration of glycolysis and TCA cycle metabolites responsive to oxidative stress. Error bars represent 1 S.E.
(TIF)

S6 Fig Relative concentration of glutathione and glutathione disulfide. Error bars represent 1 S.E.
(TIF)

S1 File Excel spreadsheet containing metabolite data normalized to optical density of cultures. (XLSX)

S2 File Excel spreadsheet containing lipid data normalized to optical density of cultures. (XLSX)
