# Supplementary material for: Microcystin-LR does not induce alterations to transcriptomic or metabolomic profiles of a model heterotrophic bacterium
Source: PLoS One. 2017 Dec 14;12(12):e0189608. doi: 10.1371/journal.pone.0189608 (PMC5730168; doi:10.1371/journal.pone.0189608)
Supplement: S1 Table — (DOCX) [file pone.0189608.s001.docx]

| Supplemental Table 1. Summary of RNA-seq libraries. | | | | |  |  |  |
| --- | --- | --- | --- | --- | --- | --- | --- |
|  |  | Time |  |  |  | Reads Uniquely | Percent of Total |
| Sample |  | Point |  | Total | Non-ribosomal | Mapped to | Reads Mapped to |
| Library | Treatment | (min.) | Replicate | Reads | Reads | Protein Genes | Protein Genes |
| 21 | 1 mg/L | 0 | 1 | 1,856,952 | 578,629 | 436,607 | 23.5% |
| 36 | 1 mg/L | 0 | 2 | 2,628,949 | 330,742 | 216,580 | 8.2% |
| 51 | 1 mg/L | 0 | 3 | 2,794,327 | 627,730 | 426,142 | 15.3% |
| 22 | 1 mg/L | 15 | 1 | 1,648,938 | 469,134 | 370,750 | 22.5% |
| 37 | 1 mg/L | 15 | 2 | 2,778,731 | 375,855 | 257,253 | 9.3% |
| 52 | 1 mg/L | 15 | 3 | 2,261,390 | 378,607 | 214,136 | 9.5% |
| 23 | 1 mg/L | 30 | 1 | 2,102,583 | 558,751 | 432,730 | 20.6% |
| 38 | 1 mg/L | 30 | 2 | 2,632,036 | 378,221 | 243,783 | 9.3% |
| 53 | 1 mg/L | 30 | 3 | 2,379,060 | 564,473 | 450,751 | 18.9% |
| 24 | 1 mg/L | 45 | 1 | 2,401,840 | 538,169 | 440,542 | 18.3% |
| 39 | 1 mg/L | 45 | 2 | 3,000,842 | 495,302 | 370,282 | 12.3% |
| 54 | 1 mg/L | 45 | 3 | 2,090,163 | 366,204 | 264,066 | 12.6% |
| 25 | 1 mg/L | 60 | 1 | 674,463 | 135,264 | 94,693 | 14.0% |
| 40 | 1 mg/L | 60 | 2 | 2,286,284 | 374,551 | 259,111 | 11.3% |
| 55 | 1 mg/L | 60 | 3 | 1,620,608 | 297,337 | 189,717 | 11.7% |
| 16 | 10 mg/L | 0 | 1 | 1,931,743 | 556,205 | 427,763 | 22.1% |
| 31 | 10 mg/L | 0 | 2 | 2,566,695 | 296,977 | 183,825 | 7.2% |
| 46 | 10 mg/L | 0 | 3 | 1,840,448 | 356,666 | 247,832 | 13.5% |
| 17 | 10 mg/L | 15 | 1 | 2,424,433 | 744,250 | 609,503 | 25.1% |
| 32 | 10 mg/L | 15 | 2 | 1,666,033 | 301,110 | 220,880 | 13.3% |
| 47 | 10 mg/L | 15 | 3 | 1,845,845 | 269,194 | 167,967 | 9.1% |
| 18 | 10 mg/L | 30 | 1 | 2,363,228 | 594,756 | 481,984 | 20.4% |
| 33 | 10 mg/L | 30 | 2 | 2,045,538 | 333,893 | 227,957 | 11.1% |
| 48 | 10 mg/L | 30 | 3 | 1,832,610 | 324,590 | 210,914 | 11.5% |
| 19 | 10 mg/L | 45 | 1 | 1,754,863 | 454,617 | 363,914 | 20.7% |
| 34 | 10 mg/L | 45 | 2 | 1,608,849 | 308,057 | 214,959 | 13.4% |
| 49 | 10 mg/L | 45 | 3 | 1,453,203 | 194,728 | 140,580 | 9.7% |
| 20 | 10 mg/L | 60 | 1 | 1,957,958 | 349,417 | 271,909 | 13.9% |
| 35 | 10 mg/L | 60 | 2 | 1,516,127 | 368,479 | 256,819 | 16.9% |
| 50 | 10 mg/L | 60 | 3 | 2,521,679 | 576,184 | 461,644 | 18.3% |
| 26 | Control | 0 | 1 | 2,727,789 | 526,879 | 394,871 | 14.5% |
| 41 | Control | 0 | 2 | 2,179,753 | 278,043 | 181,110 | 8.3% |
| 56 | Control | 0 | 3 | 2,241,147 | 373,661 | 230,825 | 10.3% |
| 27 | Control | 15 | 1 | 1,995,203 | 437,396 | 322,762 | 16.2% |
| 42 | Control | 15 | 2 | 2,874,844 | 433,020 | 283,312 | 9.9% |
| 57 | Control | 15 | 3 | 1,912,173 | 325,032 | 193,720 | 10.1% |
| 28 | Control | 30 | 1 | 1,609,873 | 308,325 | 224,940 | 14.0% |
| 43 | Control | 30 | 2 | 2,393,595 | 268,645 | 163,755 | 6.8% |
| 58 | Control | 30 | 3 | 2,154,967 | 335,083 | 219,299 | 10.2% |
| 29 | Control | 45 | 1 | 2,495,145 | 480,192 | 352,343 | 14.1% |
| 44 | Control | 45 | 2 | 1,750,453 | 374,993 | 252,248 | 14.4% |
| 59 | Control | 45 | 3 | 1,632,540 | 259,588 | 165,363 | 10.1% |
| 30 | Control | 60 | 1 | 2,359,523 | 533,515 | 405,544 | 17.2% |
| 45 | Control | 60 | 2 | 2,270,199 | 494,089 | 363,243 | 16.0% |
| 60 | Control | 60 | 3 | 2,451,272 | 424,981 | 275,484 | 11.2% |
| Total |  |  |  | 95,534,894 | 18,351,534 | 13,184,412 |  |
| Mean |  |  |  | 2,122,998 | 407,812 | 292,987 | 13.8% |
