# Supplementary figures and images for: Microcystin-LR does not induce alterations to transcriptomic or metabolomic profiles of a model heterotrophic bacterium
Source: PLoS One. 2017 Dec 14;12(12):e0189608. doi: 10.1371/journal.pone.0189608 (PMC5730168; doi:10.1371/journal.pone.0189608)

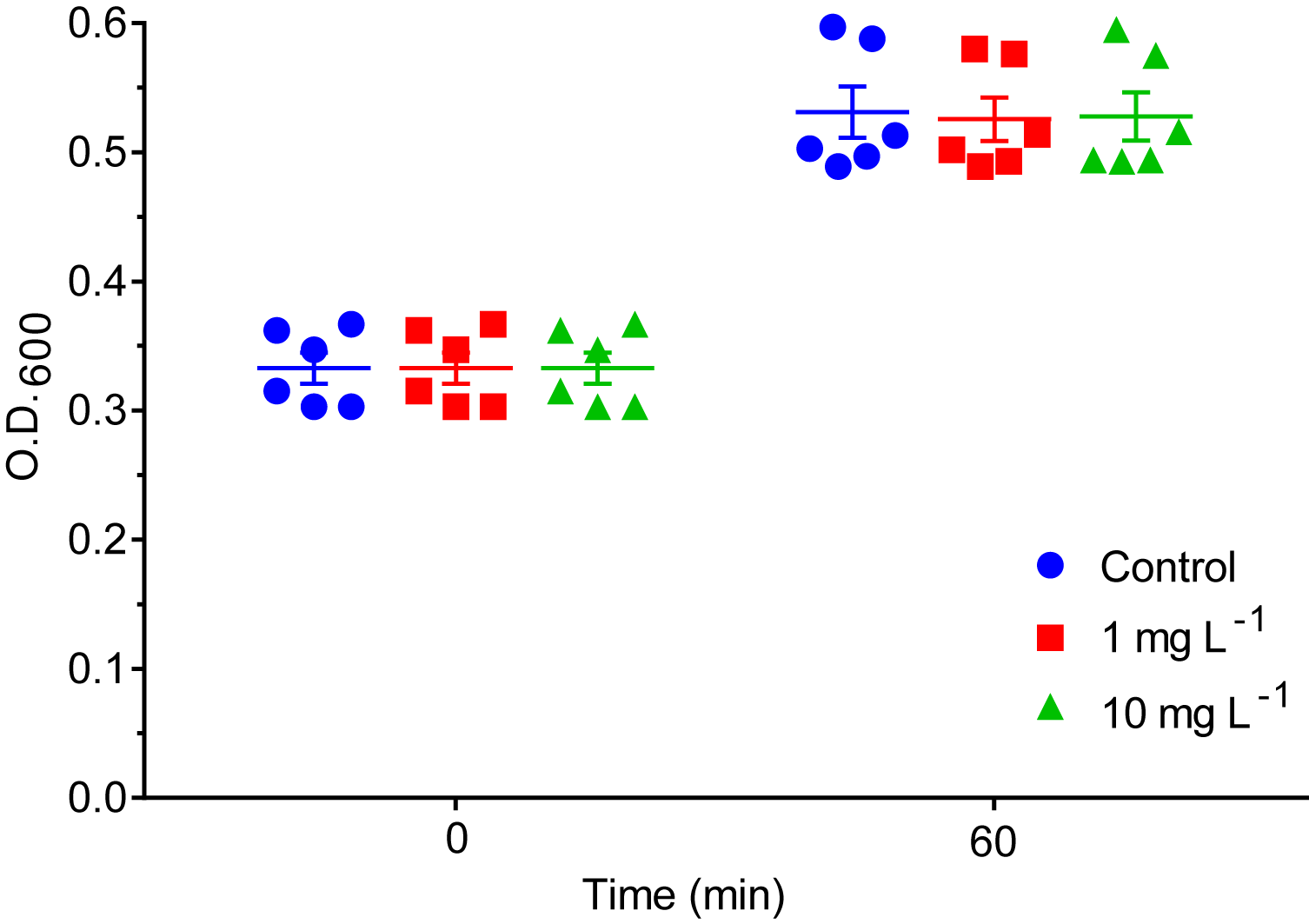

Supplement: S1 Fig — O.D.600 measurements of cultures at the start (time = 0) and end (time = 60 min) of treatments. Symbols represent actual data points of biological replicates. The horizontal bars represent the mean. (TIF) [file pone.0189608.s002.tif]

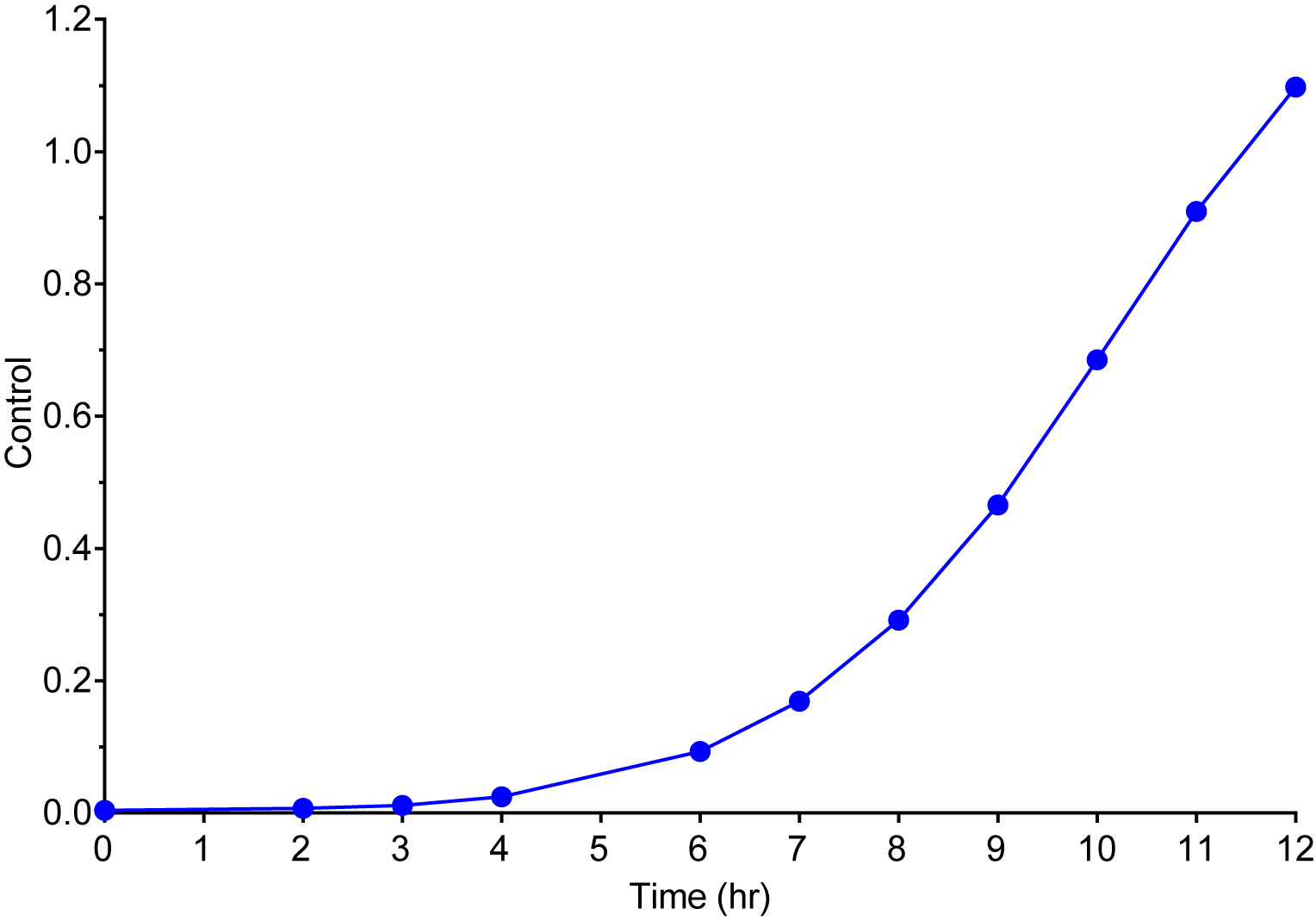

Supplement: S2 Fig — Growth curves of E. coli in M9 minimal medium with addition of 4 g L-1 glucose and 1 mg L-1 thiamine HCl. These curves were generated in preliminary experiments and represent conditions identical to those of the master cultures described in Methods. Treatments were imposed on cultures approximately 8 hr after inoculation when O.D.600 was ~0.3. Error bars represent 1 S.E., but are smaller than symbol size. Growth rates calculated from these curves were identical (μ = 0.47 hr-1) to those calculated from the control cultures during the experiment. n = 2. (TIF) [file pone.0189608.s003.tif]

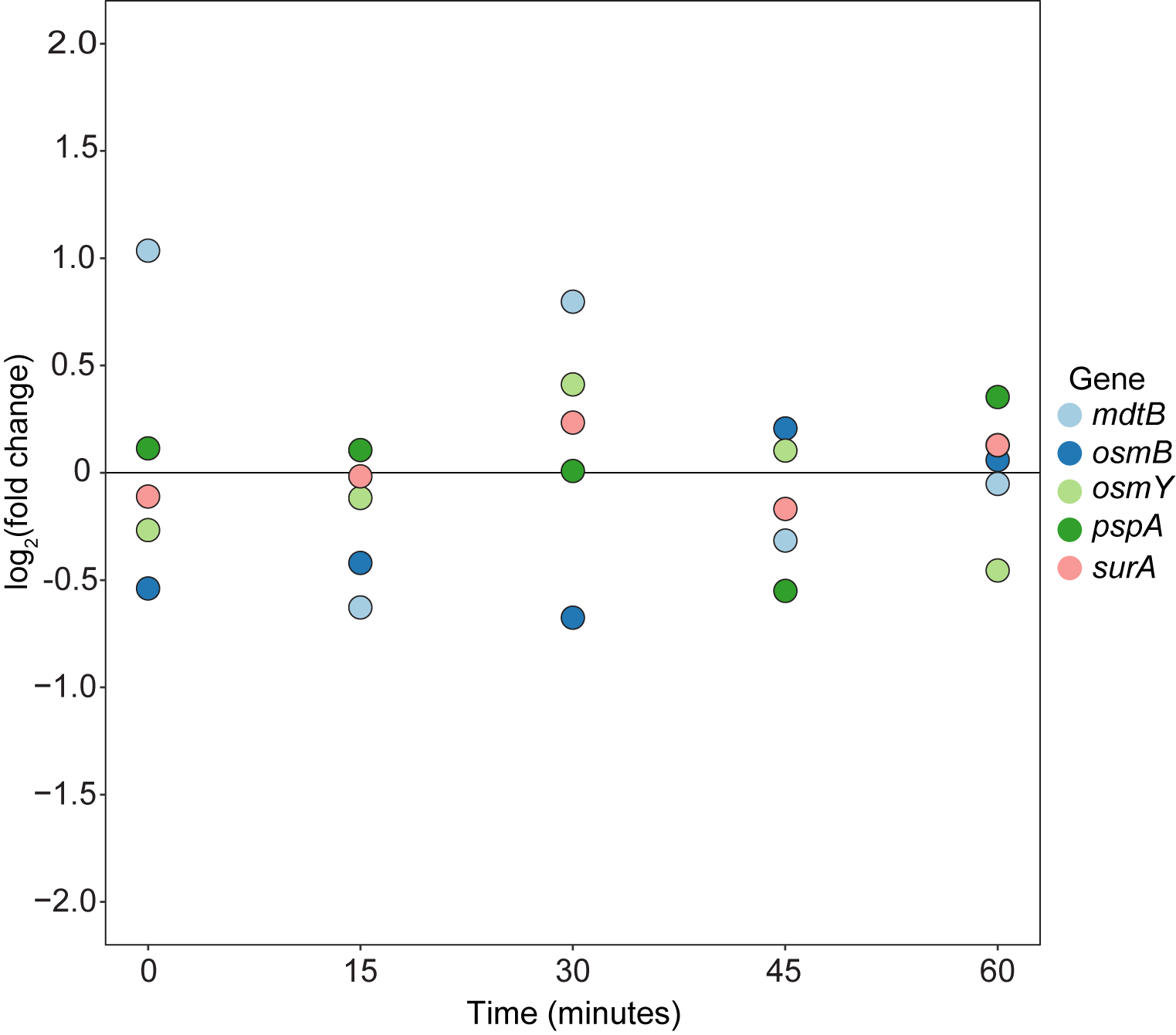

Supplement: S3 Fig — Each point represents the log2 fold change relative to control at a given time point. The horizontal line at 0 represents equal expression in treatment and control. Gene abbreviations: Bae regulon: mdtB (multidrug efflux pump RND permease subunit). Psp regulon: pspA (phage shock protein A). Rcs regulon: osmB (osmotically and stress inducible lipoprotein), osmY (osmotically inducible periplasmic chaperone), surA (periplasmic OM porin chaperone). (TIF) [file pone.0189608.s004.tif]

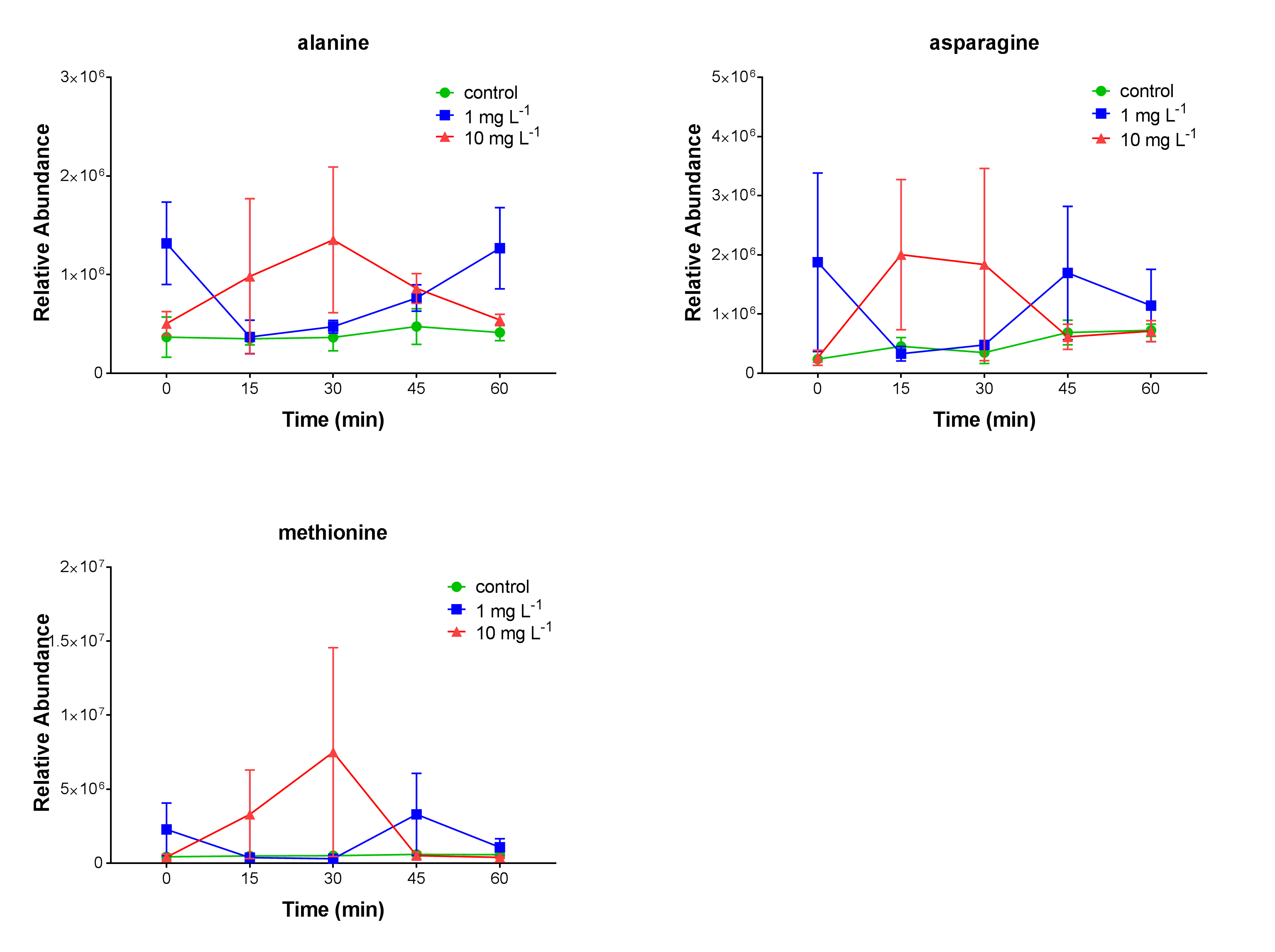

Supplement: S4 Fig — Error bars represent 1 S.E. (TIF) [file pone.0189608.s005.tif]

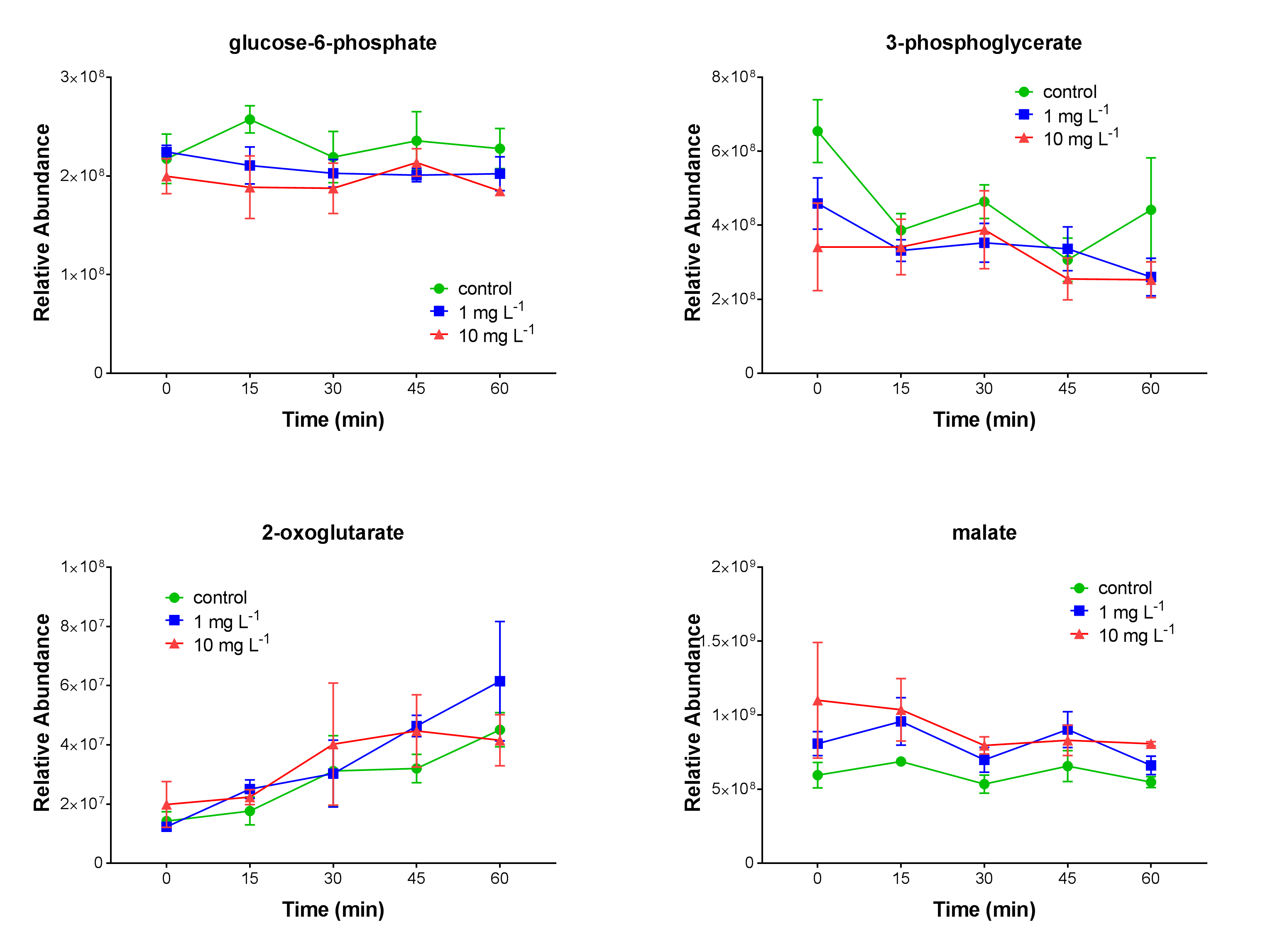

Supplement: S5 Fig — Error bars represent 1 S.E. (TIF) [file pone.0189608.s006.tif]

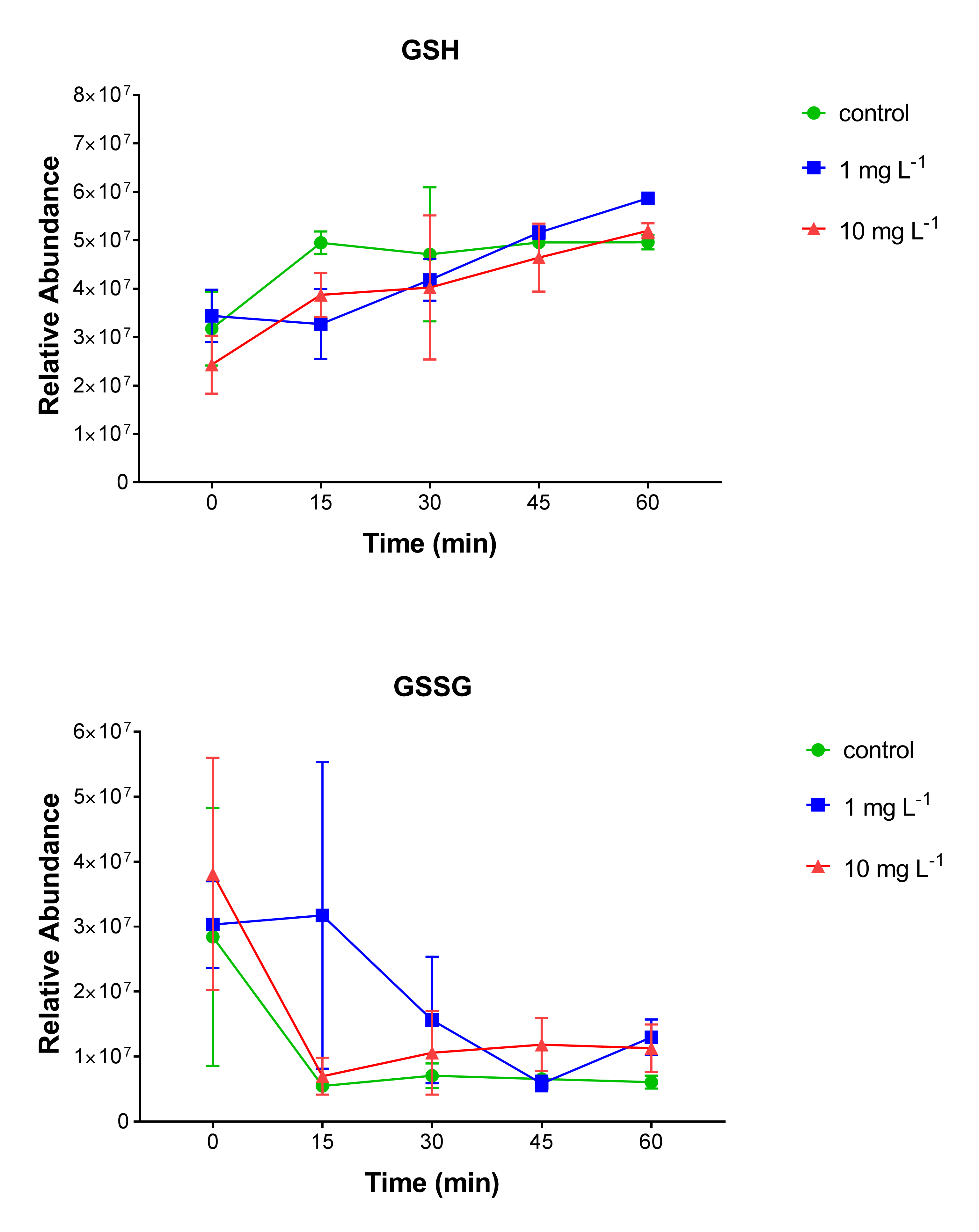

Supplement: S6 Fig — Error bars represent 1 S.E. (TIF) [file pone.0189608.s007.tif]
